# Bentracimab for Surgical and Perioperative Bleeding: Clinical Efficacy, Emerging Safety Concerns, and Implementation Challenges (Cost, Accessibility, and Evidence Gaps)

**DOI:** 10.7759/cureus.108189

**Published:** 2026-05-03

**Authors:** Anisha Bai, Waseem Abbas, Faiq Baig, Danish Kumar, Vanish Kumar, Muhammad Mudasir, Ashir Kanwal, Haroon Chaudhry, Victoria L Reimer

**Affiliations:** 1 Medicine, Liaquat University of Medical and Health Sciences, Jamshoro, PAK; 2 Critical Care, Thomas Jefferson University Hospital, Sewell, USA; 3 Pulmonary/Critica Care, Thomas Jefferson University Hospital, Sewell, USA

**Keywords:** bentracimab, emergency surgery, hemorrhage management, monoclonal antibody, p2y12 inhibitors, perioperative bleeding, platelet inhibition, surgical hemostasis, ticagrelor reversal

## Abstract

Perioperative and surgical bleeding in patients on ticagrelor therapy remains a clinical challenge, with many patients undergoing urgent procedures experiencing severe bleeding. Ticagrelor is widely used in cardiovascular disease management. A notable proportion of patients undergoing percutaneous coronary intervention (PCI) later require surgical procedures within two years, creating significant challenges in perioperative care, including increased morbidity, the need for transfusions, and delays in life-saving surgery. These challenges are related to the reversible nature of P2Y₁₂ inhibition by ticagrelor and the presence of its circulating active metabolite. Together, these factors contribute to sustained platelet inhibition and reduce the effectiveness of non-specific reversal strategies. Platelet transfusion and supportive care have shown limited effectiveness, largely due to rapid re-inhibition of transfused platelets by circulating ticagrelor and its active metabolite. Bentracimab (PB2452), a high-affinity humanized monoclonal antibody fragment, was developed to bind ticagrelor and its active metabolite, thereby reversing platelet inhibition. This narrative review integrates available preclinical, translational, and clinical data assessing the efficacy, safety, and perioperative use of bentracimab in ticagrelor-associated bleeding in the setting of urgent surgery. In early-phase studies and the pivotal phase 3 REVERSE-IT trial, bentracimab rapidly restored platelet function, with effects observed within minutes and sustained for up to 24 hours. Pharmacodynamic reversal of platelet inhibition was consistent. Clinical outcomes, such as effective hemostasis, were achieved in a high proportion of patients, as reported in most available studies, supporting its potential use in urgent surgical settings. Short-term safety profiles appear promising, with low rates of infusion reactions and no clear evidence of prothrombotic effects in early-phase trials. However, thrombotic events have been observed in high-risk clinical scenarios, although these have not been definitively attributed to bentracimab. This warrants cautious interpretation, given limited follow-up and the absence of comparator trials. Current evidence is derived mainly from single-arm and early-phase studies. Randomized comparative data are lacking, and long-term safety, including thrombotic risk after reversal, remains unclear. Additional challenges include cost, access, and integration into perioperative care pathways. Compared to non-specific reversal strategies such as platelet transfusion and supportive care, bentracimab offers rapid and targeted neutralization of ticagrelor, with restoration of platelet function. Overall, bentracimab shows promising potential for integration into surgical and perioperative bleeding management protocols, pending further comparative studies and long-term outcome data.

## Introduction and background

Perioperative and surgical bleeding events continue to pose a significant clinical problem, especially in patients on potent antiplatelet therapy for the management of cardiovascular disease. Ticagrelor is a direct and reversible P2Y₁₂ receptor blocker widely used in acute coronary syndrome and high-risk cardiovascular interventions due to its rapid onset and potent antithrombotic effects compared with previously used medications such as clopidogrel and prasugrel. Despite these pharmacological advantages, the risk of major bleeding during urgent surgery and life-threatening situations remains a significant clinical challenge, where timely reversal of platelet inhibition is required [1-3]. In routine clinical practice, ticagrelor-associated bleeding accounts for approximately 41% of adverse events, which results in premature discontinuation in roughly 17% of treated patients [4].

Historically, ticagrelor did not have a specific reversal agent, unlike anticoagulants such as direct oral anticoagulants and vitamin K antagonists, which have established reversal strategies. Conventional approaches such as platelet transfusion, desmopressin, or supportive hemostatic measures have shown inconsistent effectiveness. Because ticagrelor and its active metabolite have longer half-lives than aspirin (approximately 9 and 12 hours, respectively), restoration of platelet function is delayed. Even after the drug is cleared from circulation, a substantial number of functional platelets may still be required within the first 48 hours. The effectiveness of platelet transfusion in reversing ticagrelor’s antiplatelet effect depends on both the time elapsed since the last dose and the number of platelets administered. Within the first 24 hours, transfusion has minimal impact; however, between 24 and 48 hours, administration of 2-3 units can significantly restore platelet function [5,6]. This has necessitated interim management strategies for perioperative bleeding in ticagrelor-treated patients, which are often associated with delayed surgery, increased transfusion requirements, and thrombotic risk due to premature discontinuation of antiplatelet therapy.

Bentracimab (PB2452) (PhaseBio Pharmaceuticals, Malvern, PA, USA) is an anti-ticagrelor humanized monoclonal antibody fragment designed to neutralize ticagrelor and its active metabolite. Early-phase studies and subsequent clinical investigations have demonstrated rapid restoration of platelet function following bentracimab administration, with effects observed within minutes and sustained for clinically relevant durations [7]. These characteristics suggest that bentracimab may be a promising targeted reversal agent for use in emergent scenarios associated with a high risk of significant bleeding.

Clinical interest in bentracimab continues to grow. Existing data are mostly based on early trials and interim analyses, and long-term follow-up data remain limited, with no randomized comparator trials currently available. While short-term safety appears favorable, important questions persist regarding thrombotic risk following ticagrelor reversal, optimal perioperative integration, cost considerations, and efficacy relative to nonspecific reversal strategies and other antithrombotic antidotes [8]. Previous literature reviews have primarily focused on pharmacologic mechanisms or provided generalized summaries of clinical trial findings. In contrast, this narrative review aims to integrate pharmacologic, clinical, and perioperative perspectives, with a specific focus on practical perioperative management strategies, safety considerations, and cross-specialty applicability.

## Review

Methods⁠

This study was conducted as a narrative review to synthesize current evidence on the safety, pharmacology, and perioperative clinical utility of bentracimab in patients with ticagrelor-associated bleeding. A narrative approach was selected to enable contextual interpretation of heterogeneous clinical and translational data rather than formal quantitative synthesis.

A literature search was performed using PubMed/MEDLINE, Embase, Cochrane Library, and Google Scholar, and was restricted to studies published from 2016 to March 2026, as this timeframe captures the period during which bentracimab entered preclinical and clinical investigation and ensures inclusion of the most relevant and contemporary evidence. Search terms focused on bentracimab (PB2452), ticagrelor reversal, antiplatelet reversal strategies, perioperative bleeding, urgent surgery, and hemostatic management. An example search strategy used in PubMed/MEDLINE was (“bentracimab” OR “PB2452”) AND (“ticagrelor reversal” OR “P2Y₁₂ inhibitor reversal”) AND (“perioperative bleeding” OR “urgent surgery” OR “hemostatic management”).

To ensure cohesion and integrity of the narrative review, studies published in languages other than English were excluded, along with conference abstracts, to minimize potential translation bias and misinterpretation of scientific data. Although formal inclusion and exclusion criteria were not predefined, studies were selected based on broad relevance to bentracimab pharmacology, clinical efficacy, safety outcomes, and perioperative application. Priority was given to clinical trials, translational studies, and high-quality reviews that directly addressed ticagrelor reversal. Screening was performed based on titles and abstracts, followed by full-text review, using thematic domains (pharmacologic mechanisms, clinical efficacy, safety, and perioperative application). Eligible sources included preclinical studies, clinical trials, interim analyses, observational studies, and relevant reviews. Data were synthesized qualitatively, and no meta-analysis was performed. Given the narrative design, a formal risk-of-bias assessment was not conducted; however, studies were critically appraised based on study design and methodological rigor.

Overall, this narrative design may be subject to selection bias, and the absence of predefined inclusion criteria, formal study selection flow metrics, and standardized quality assessment may limit reproducibility and interpretability. Additionally, exclusion of non-English studies may introduce language bias.

Mechanism and pharmacologic rationale

Ticagrelor is a directly acting and reversible platelet (P2Y₁₂ receptor) blocker widely used in acute coronary syndrome, coronary artery stent recipients, and other thrombotic disorders (such as ischemic stroke) due to its rapid onset and potent antithrombotic effects [9,10]. By inhibiting adenosine diphosphate (ADP)-mediated platelet activation, ticagrelor provides rapid and potent antiplatelet effects. However, both ticagrelor and its active metabolite (AR-C124910XX) remain freely circulating in plasma and interact with P2Y₁₂ receptors, leading to persistent platelet inhibition even after drug cessation. This pharmacologic property presents significant challenges during major bleeding or emergency surgical procedures, as circulating active drug can rapidly inhibit newly transfused platelets, thereby limiting the effectiveness of platelet transfusion as a reversal strategy [9,11].

Unlike irreversible thienopyridines (clopidogrel, prasugrel), the reversible binding characteristic of ticagrelor permits pharmacologic neutralization through direct drug sequestration. Bentracimab (formerly PB2452) was developed to exploit this property. It is a neutralizing recombinant human monoclonal antibody fragment (Fab) with approximately 100-fold higher binding affinity for ticagrelor and its active metabolite (AR-C124910XX) than for the platelet P2Y₁₂ receptor, based on in vitro binding studies [5,10].

**Figure 1:**
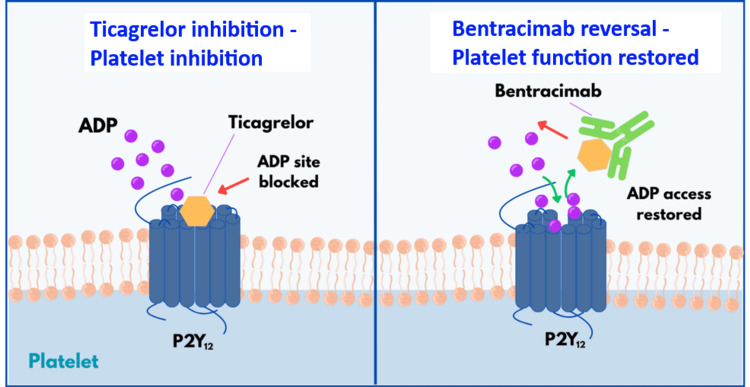
Bentracimab's mechanism of action in reversing ticagrelor-induced platelet inhibition ADP: adenosine diphosphate. Source: [8]. Image Credit: Faiq Baig (created using Canva Pro, Canva Pty Ltd., Sydney, Australia). This diagram was manually constructed to ensure logical accuracy and clinical precision.

Thus, the high-affinity, selective binding of bentracimab to ticagrelor enables rapid and efficient sequestration of unbound circulating drug, thereby providing a strong pharmacologic basis for targeted reversal of platelet inhibition without disrupting endogenous ADP-mediated platelet signaling pathways [5]. As receptor-bound ticagrelor dissociates, the absence of circulating free drug prevents rebinding, permitting restoration of ADP-mediated platelet activation and aggregation, as shown in Figure 1. Bentracimab exhibits no interaction with ADP, adenosine, or other purinergic receptors, thereby minimizing unintended off-target pharmacologic effects. The resulting drug-antibody complexes are expected to be cleared via renal excretion following degradation, although direct human pharmacokinetic characterization of this pathway remains limited [5].

Pharmacokinetic-pharmacodynamic modeling suggests that intravenous administration as a bolus followed by infusion produces rapid reversal within minutes and sustains normalization of platelet function across multiple assays for 20-24 hours [5]. Importantly, while bentracimab demonstrates high-affinity binding to ticagrelor, binding kinetics alone do not fully account for clinical reversibility, which is primarily driven by a reduction in unbound (pharmacologically active) drug concentrations. These findings support weight- and exposure-based dosing regimens that may provide optimal and sustained reversal in acute bleeding or emergent surgical settings. Moreover, clinical and translational data support the pharmacologic basis for the use of bentracimab in achieving selective reversal of ticagrelor. Because it directly targets circulating drugs rather than attempting to overcome receptor blockade, bentracimab offers a mechanism-based approach to hemostasis specifically in ticagrelor-exposed patients [5,11].

Clinical evidence for hemostatic reversal

The management of life-threatening hemorrhagic complications and the need for emergent surgical intervention in patients taking ticagrelor pose significant clinical challenges. Ticagrelor is a potent, reversible P2Y₁₂ receptor antagonist widely used in patients with a history of thrombotic events, such as acute coronary syndrome and stroke, due to its greater antiplatelet efficacy compared with clopidogrel. However, this pharmacological profile complicates bleeding management. The drug binds reversibly and has a circulating active metabolite, making traditional nonspecific therapies (such as platelet transfusion) less effective [12,13]. Despite ticagrelor’s clinical benefits, an increased bleeding risk has consistently emerged as a key limitation of its use. These limitations provide a strong clinical rationale for the development of targeted hemostatic reversal agents, such as bentracimab.

Summary of Preclinical and Early Clinical Studies

Preclinical trials have shown that bentracimab binds both circulating and receptor-bound ticagrelor and its metabolites, resulting in rapid neutralization of ticagrelor-induced platelet inhibition in vitro and near-complete restoration of platelet aggregation in animal models of bleeding, without inducing excessive platelet activation or prothrombotic effects [8]. Table 1 presents a summary of these trials.

**Table 1:** Summary of clinical trials PRU: platelet reaction unit.

Study	Study design	Participants; n/gender	Study duration	Dose evaluated	Key findings
ClinicalTrials.gov [11]	Randomized, double-blind, placebo-controlled	64 healthy volunteers (~75% male)	~48 hours + follow-up	Single IV infusion up to 18 g	Near-complete reversal within 5 min; sustained >20 h; no rebound platelet activity
ClinicalTrials.gov [14]	Randomized, double-blind, placebo-controlled, dose-escalation, pharmacodynamic study	48 older healthy subjects (~70% male)	~72 hours	Escalating IV doses 6-18 g	Clear dose-dependent effect; higher doses produced rapid and durable reversal
ClinicalTrials.gov [15]	Randomized, double-blind, placebo-controlled	90 participants (50-80 years) (~68% male)	~7 days	IV 9-18 g	PRU restored within 5-10 min, sustained 24 h even with aspirin
ClinicalTrials.gov [16]	Open-label, single-arm, multicenter	150 patients (~72% male)	35-day follow-up	IV bolus + infusion 18-36 g	In this single-arm study without comparator, 98.4% achieved effective hemostasis; rapid reversal in bleeding and surgery; enabled urgent procedures

These studies supported early-phase clinical evaluation, in which platelet function recovery was repeatedly observed within 5-10 minutes of intravenous administration. This rapid onset was reproducible across healthy volunteers, elderly participants, and patients receiving dual antiplatelet therapy. However, most studies were conducted in controlled settings and relatively stable populations, which may limit generalizability to patients with active bleeding or critical illness. Importantly, the reversal effect was durable, persisting for 20-24 hours across multiple studies and platelet function assays, with no evidence of rebound platelet hyperactivity [11,14-16].

Early-phase trials established a clear and reproducible dose-response relationship, confirming the pharmacologic precision of bentracimab. In the randomized phase 1 trial involving ticagrelor-pretreated healthy volunteers, escalating doses of bentracimab produced progressively greater and consistent platelet function recovery, with near-complete reversal achieved at higher dose levels [11]. This dose-dependent effect was sustained for more than 20 hours and occurred without serious adverse events, thrombotic complications, or rebound platelet activation in the studied populations.

In the phase 2a trial, bentracimab demonstrated rapid and sustained normalization of platelet function in approximately 90% of participants across two study parts. Part A showed safety and efficacy in older and elderly individuals receiving dual antiplatelet therapy with ticagrelor and aspirin, while Part B confirmed effective reversal in younger participants pretreated with ticagrelor alone [14]. Although these findings supported the use of fixed-dose regimens in subsequent studies, they are primarily based on pharmacodynamic endpoints rather than clinical outcomes. Collectively, these results enabled subsequent trials to adopt fixed-dose regimens with confidence, eliminating the need for dose titration or laboratory-guided adjustment. Overall, the data indicate that bentracimab provides predictable, scalable, and durable neutralization of ticagrelor based on well-defined pharmacodynamic principles.

A critical advancement demonstrated by the clinical development program is the successful translation of pharmacologic reversal into clinically meaningful hemostasis. While early trials in healthy volunteers established proof of mechanism and safety, later studies evaluated bentracimab in populations with substantially higher bleeding risk. In the phase 2b trial involving elderly participants pretreated with ticagrelor with or without aspirin, rapid platelet function reversal was achieved in 95% of participants, typically within 5-10 minutes, and was sustained for up to 24 hours without rebound inhibition or prothrombotic events [15].

This translational consistency was confirmed in the pivotal phase 3 REVERSE-IT trial, which enrolled patients with life-threatening bleeding or those requiring urgent surgical or invasive procedures [16]. In this real-world setting, bentracimab produced rapid increases in platelet reactivity units and was associated with effective hemostasis in approximately 98% of patients, as defined by prespecified clinical criteria (e.g., adjudicated bleeding control or intraoperative hemostatic assessment). Surgical interventions were performed safely, with adequate intraoperative hemostasis in over 90% of cases and minimal transfusion requirements [16]. However, the single-arm design substantially limits causal inference, as outcomes cannot be directly attributed to bentracimab in the absence of a control group. Furthermore, without a comparator, improvements relative to standard care remain inferential rather than definitively established. While these findings are encouraging, their interpretation should be contextualized by the lack of randomization, potential selection bias, and limited data on hard clinical endpoints such as mortality.

Collectively, available evidence indicates that bentracimab produces rapid and sustained reversal of ticagrelor-induced platelet inhibition, with pharmacodynamic effects that appear to translate into clinically meaningful hemostasis in selected populations. Nevertheless, conclusions regarding consistency, reliability, and broad generalizability should be made with caution, given that much of the evidence is derived from controlled early-phase studies and non-randomized clinical data.

Safety profile

The short-term safety of bentracimab (PB2452) has been evaluated initially in early-phase trials involving healthy volunteers and subsequently in open-label studies in patients with major bleeding or those undergoing urgent surgical procedures while receiving ticagrelor. Table 2 summarizes the strength of evidence related to the safety and risk domains of bentracimab.

**Table 2:** Summary of the strength of evidence for safety and risk domains of bentracimab

Study	Study design	Safety/risk domain addressed	Study duration	Key findings	Strength of evidence	Remaining uncertainties
ClinicalTrials.gov [11]	Randomized, double-blind, placebo-controlled	Short-term safety, acute thrombotic risk	~48 hours + follow-up	Near-complete reversal within 5 min; sustained >20 h; no rebound platelet activity	Controlled study in healthy volunteers; consistent rapid platelet reversal and absence of thrombosis or rebound across participants	Small sample size limits detection of rare adverse events; healthy volunteers may not reflect bleeding-prone or comorbid populations
ClinicalTrials.gov [14]	Randomized, double-blind, placebo-controlled, dose-escalation, pharmacodynamic study	Dose-response safety, age-related risk	~72 hours	Clear dose-dependent effect; higher doses produced rapid and durable reversal	Consistent reversal in the elderly population; limited by short follow-up and absence of active bleeding	Short-term follow-up; patients not actively bleeding, limiting extrapolation to high-risk clinical scenarios
ClinicalTrials.gov [15]	Randomized, double-blind, placebo-controlled	Safety in higher-risk population	~7 days	PRU restored within 5-10 min, sustained 24 h even with aspirin	Outcomes consistent, but the trial was not powered to detect rare thrombotic events; sample size modest	Limited number of participants; cannot exclude uncommon thrombotic events or delayed adverse effects; lacks a real-world bleeding context
ClinicalTrials.gov [16]	Open-label, single-arm, multicenter	Safety in real-world bleeding and surgical patients	35-day follow-up	98.4% achieved effective hemostasis; rapid reversal in bleeding and surgery; enabled urgent procedures	Consistent results across urgent procedures and bleeding contexts; short-term follow-up only	Long-term outcomes have not been fully assessed

In the first-in-human phase I randomized, placebo-controlled trial (NCT03492385), gradually escalated intravenous doses of bentracimab were administered to ticagrelor-pretreated healthy volunteers and evaluated for short-term safety, with no evidence of dose-limiting toxicities, treatment-related deaths, or serious infusion-related reactions [11]. Although the incidence of adverse events was higher among PB2452 recipients compared with placebo, these events were predominantly mild and transient, consisting mainly of infusion-site reactions and minor procedural complaints [3,11]. Subsequent evaluation in older adult volunteers in a Phase 2a trial (NCT03928353) further confirmed the short-term tolerability of bentracimab. Overall, bentracimab has demonstrated favorable safety outcomes, with no significant adverse events or thrombotic complications reported during the observation period. Following ticagrelor reversal, no platelet hyperreactivity was observed, supporting an overall favorable safety profile, with no evidence of key theoretical concerns associated with rapid neutralization of potent antiplatelet therapy. These findings indicate that bentracimab can be administered safely in controlled clinical settings without signs of immediate prothrombotic or systemic toxicity [14]. More clinically relevant safety data have emerged from the phase 3 REVERSE-IT trial (NCT04286438), an ongoing multicenter, open-label study enrolling patients with life-threatening bleeding or requiring urgent surgical intervention. Interim and published analyses demonstrated acceptable tolerability in these acutely ill populations, with no serious allergic reactions or treatment-related deaths attributed to the study drug. Reported serious adverse events were not linked to bentracimab administration but rather to the underlying severity of illness. Taken together with data from controlled volunteer studies, these findings suggest a consistent and favorable short-term safety profile for PB2452 across diverse clinical settings [16,17].

One of the major concerns with antiplatelet reversal agents is the risk of thrombotic events following restoration of platelet function, particularly in patients with underlying cardiovascular disease. Early-phase clinical trials are reassuring in this regard, as no thrombotic events were reported during short-term follow-up in Phase 1 and 2 studies (NCT03492385 and NCT03928353) after bentracimab administration. It is noteworthy that these studies were conducted in highly selected populations with low baseline thrombotic risk, which may limit generalizability to higher-risk patients [11,14]. In the phase 3 REVERSE-IT trial (NCT04286438), thrombotic events were infrequent, occurring in approximately 5% of patients, and were observed predominantly in the early post-reversal period. These events occurred in the context of acute clinical scenarios (including active bleeding, urgent surgery, or temporary interruption of antithrombotic therapy) that independently increase thrombotic risk. Given the single-arm design without a comparator group, thrombotic events reported during the peri-treatment period cannot be definitively attributed to bentracimab, and their incidence should be interpreted in the context of the inherently high-risk population [16,17].

Current evidence suggests that while PB2452 rapidly restores platelet function, the incidence of clinically significant thrombotic events remains relatively low, particularly in controlled settings. Nevertheless, the absence of a comparator arm in the REVERSE-IT trial and the limited dataset mean that residual uncertainty persists regarding post-reversal thrombosis, especially in patients with recent acute coronary syndromes or complex cardiovascular comorbidities. Ongoing surveillance and post-marketing studies will be essential to fully delineate the thrombotic risk profile of bentracimab in real-world practice [16].

Although bentracimab demonstrates a favorable short-term safety profile, no clear long-term safety data are currently available. Evaluation has been limited to hours and days following administration, with follow-up durations of up to 35 days [11,14,15]. As such, there is no definitive evidence regarding long-term adverse effects associated with bentracimab, including delayed thrombotic complications, immunologic reactions, or effects related to repeated exposure.

Anti-drug antibodies (ADA) have been detected in a subset of participants across early-phase clinical trials and the phase 3 REVERSE-IT trial [11,14-16]. Importantly, ADA development was not associated with reduced pharmacodynamic efficacy, hypersensitivity reactions, or acute safety signals during short-term follow-up [11,14-16]. However, the clinical implications remain uncertain, particularly regarding repeated exposure, delayed immune-mediated effects, and long-term safety, as extended follow-up data are not yet available. Therefore, while routine monitoring was not indicated in the reported studies, continued post-marketing surveillance and long-term immunogenicity assessment are warranted, especially in patients who may require repeated administration [16].

Nevertheless, the clinical significance of these findings over extended periods, particularly in patients requiring repeated reversal or chronic ticagrelor therapy, remains unknown. Furthermore, there are currently no randomized controlled trials or post-marketing surveillance data evaluating long-term outcomes, rare adverse events, or delayed complications [16,17]. This lack of long-term safety data represents a major knowledge gap that must be addressed to fully characterize the risk-benefit profile of bentracimab across diverse patient populations. Overall, bentracimab appears effective; however, its long-term safety remains uncertain, limiting definitive conclusions regarding widespread clinical use.

Perioperative​ clinical application and decision-making‌

The greatest perioperative challenges in ticagrelor therapy arise in the management of severe bleeding or surgical emergencies. The reduced effectiveness of standard treatment strategies, such as platelet transfusion, is due to ticagrelor’s active metabolite and its potent, reversible P2Y₁₂ inhibition, which necessitates a prolonged drug washout period before surgery. Bentracimab is a newly developed targeted therapy designed to rapidly restore platelet function in potentially life-threatening situations, based on emerging clinical trial data in the absence of formal guideline recommendations.

Patients on ticagrelor who present with life-threatening or uncontrolled bleeding, such as intracranial hemorrhage, severe gastrointestinal bleeding, or bleeding associated with hemodynamic instability, may be considered for bentracimab administration. Delaying intervention to allow spontaneous drug clearance is often impractical in these scenarios. Data from the phase 3 REVERSE-IT trial demonstrated that bentracimab rapidly reversed ticagrelor-induced platelet inhibition within minutes, with effects sustained beyond 24 hours [16]. This pharmacodynamic reversal supports its use as an escalation strategy when conventional supportive measures are inadequate [18], although current evidence is primarily derived from early-phase trials and single-arm studies.

Another critical application of bentracimab is in emergency surgery. Patients on ticagrelor may require unscheduled procedures such as trauma surgery, neurosurgery, or cardiac surgery, where waiting for a 3-5-day washout period is not feasible. In the REVERSE-IT trial, patients requiring invasive procedures or urgent surgery achieved successful hemostasis and rapid restoration of platelet function in nearly all cases. However, these findings should be interpreted cautiously, given the small subgroup size and absence of a control group [16,18]. Overall, these results suggest that bentracimab may serve as a perioperative bridging strategy in situations requiring urgent bleeding control with reduced bleeding complications.

To maintain continuous ticagrelor neutralization, clinical trials have evaluated a standardized intravenous regimen consisting of an initial bolus followed by a 16-hour infusion [16]. This approach accounts for drug distribution kinetics and maintains sustained reversal. Platelet function recovery is observed within 5-10 minutes of administration and is maintained throughout the infusion period [3,19]. Intravenous administration offers clear perioperative advantages and is ideally initiated immediately after recognition of urgent surgical need or active bleeding.

Perioperative decision-making may also be supported by platelet function monitoring, particularly in high-risk surgical settings. Available assays include VerifyNow P2Y₁₂, vasodilator-stimulated phosphoprotein (VASP) phosphorylation assay, and light transmission aggregometry (LTA) [17,19]. VerifyNow P2Y₁₂ is a point-of-care test that quantifies platelet reactivity in whole blood by measuring aggregation in response to ADP stimulation, reported as platelet reactivity units (PRU) [3]. Lower PRU values indicate greater platelet inhibition, whereas increases in PRU following bentracimab administration indicate recovery of platelet function [3]. The VASP phosphorylation assay assesses P2Y₁₂ receptor inhibition at the intracellular signaling level and reports a platelet reactivity index (PRI), where higher values reflect reduced P2Y₁₂ inhibition [3]. Light transmission aggregometry (LTA), the laboratory reference standard, measures platelet aggregation based on changes in light transmission in platelet-rich plasma following agonist stimulation [3]. In clinical studies of ticagrelor reversal, these assays have demonstrated normalization of platelet function within minutes of bentracimab administration, supporting its efficacy [3]. However, limited availability, lack of standardization across centers, cost, and turnaround time variability may restrict routine clinical use.

While objective assessment of platelet function recovery may assist surgical decision-making and evaluation of reversibility, it may not always be feasible in routine practice. In acute clinical settings, assessment of surgical bleeding and achievement of hemostasis remain central to patient management [20]. Effective perioperative use of bentracimab requires close multidisciplinary collaboration. Hematologists play a key role in patient selection, bleeding risk assessment, and interpretation of platelet function tests. Anesthesiologists must anticipate rapid changes in platelet activity and adjust intraoperative management accordingly. Surgeons must integrate reversal timing into operative planning, balancing bleeding and thrombotic risks. Institutional protocols integrating these disciplines may reduce delays and improve the quality of care [20].

Although bentracimab effectively reverses ticagrelor, restoration of platelet function exposes patients to thrombotic risk [20]. Importantly, bentracimab itself does not appear to have intrinsic prothrombotic activity; however, withdrawal of antiplatelet effect necessitates careful planning for timely resumption of therapy [18,21]. Antiplatelet therapy should be restarted once bleeding risk is acceptable, with timing and agent selection individualized based on cardiovascular risk, procedural factors, and clinical stability. In particular, patients with recent acute coronary syndrome or coronary stenting require careful coordination with cardiology to prevent ischemic complications during this vulnerable period [18,22]. Further clinical trials are needed to better define the role of bentracimab in perioperative management and to establish standardized clinical protocols.

**Figure 2:**
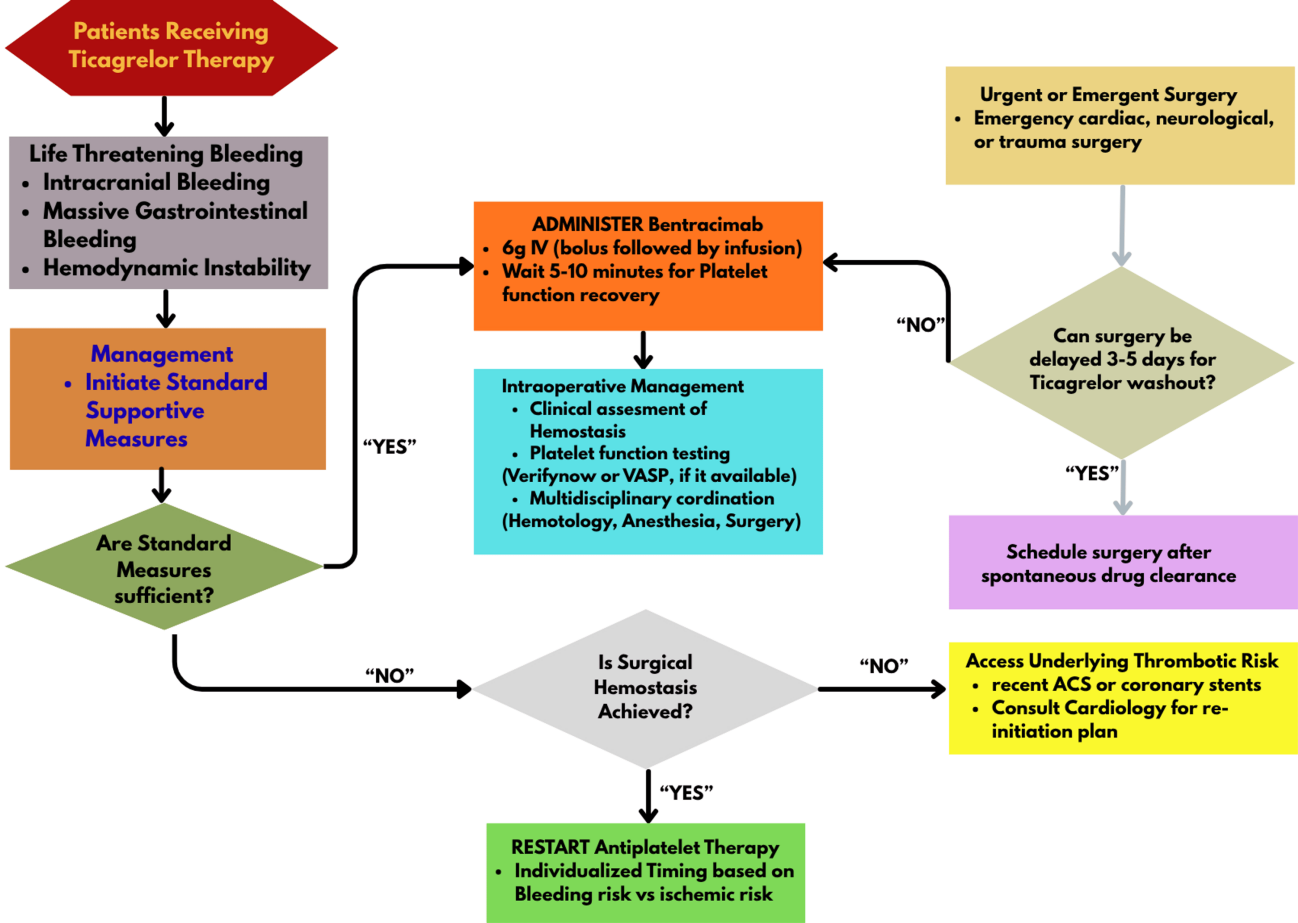
Perioperative decision-making pathway for ticagrelor reversal with bentracimab ACS: acute coronary syndrome, GI: gastrointestinal, IV: intravenous, VASP: vasodilator-stimulated phosphoprotein (phosphorylation assay). Source: [3,8,9,15,16,22]. Image Credit: Faiq Baig (created using Canva Pro, Canva Pty Ltd., Sydney, Australia). This diagram was manually constructed to ensure logical accuracy and clinical precision.

A structured perioperative decision pathway for ticagrelor reversal using bentracimab is illustrated in Figure 2.

Health system considerations and barriers

Although bentracimab represents an important advance in the targeted reversal of ticagrelor-associated bleeding, several health system-level barriers may limit its widespread adoption. Cost-effectiveness is a major concern; however, no direct economic or cost-effectiveness data for bentracimab are currently available, and existing considerations are extrapolated from other reversal agents. Experience with other targeted reversal agents, such as idarucizumab and andexanet alfa, has shown that high acquisition costs can significantly influence hospital budgets and formulary approval decisions. Real-world data demonstrate that while these agents may improve clinical outcomes, they are frequently associated with increased short-term hospital and intensive care costs, particularly in emergency and critical care settings, leading to restricted use primarily in life-threatening bleeding or urgent surgical situations [18,23]. These findings are based on other reversal agents and may not directly translate to bentracimab due to the lack of dedicated studies.

Access and logistical challenges further complicate implementation. Surveys and observational studies have shown substantial variability in the availability of reversal agents across hospitals, driven by institutional protocols, pharmacy approval processes, reimbursement concerns, and clinician familiarity. Even when supported by clinical evidence, delays in access or the absence of standardized treatment pathways can hinder timely administration, especially for agents intended for infrequent but high-acuity scenarios such as major bleeding or emergency surgery [24-26]. Experience with andexanet alfa illustrates these challenges, as its uptake has been uneven despite robust trial data, largely due to cost concerns and uncertainty regarding appropriate patient selection, providing indirect insight for emerging agents such as bentracimab [24,25].

From a guideline perspective, the incorporation of new reversal agents often lags behind clinical trial publication. Current bleeding and antithrombotic management guidelines increasingly support targeted reversal strategies but generally recommend their use in severe or life-threatening cases, reflecting both clinical benefit and economic considerations [27]. However, these recommendations do not yet specifically include bentracimab due to limited clinical and economic evidence. As real-world effectiveness and cost-effectiveness data for bentracimab continue to emerge, future guideline updates may better define its role across different clinical settings. Overall, despite its rapid and effective mechanism, cost, access, and implementation barriers are likely to restrict widespread use of bentracimab, particularly outside specialized or resource-rich centers, until more robust real-world and economic data become available.

Knowledge gaps and future directions

Although there is increasing evidence supporting the rapid reversal of ticagrelor by bentracimab, several important knowledge gaps remain that have limited its widespread application in clinical practice.

A key limitation of the current evidence base is the lack of randomized controlled trials in patients presenting with major bleeding or requiring urgent surgery. While the phase 3 REVERSE-IT trial (NCT04286438) demonstrated rapid and sustained recovery of platelet function and high rates of successful hemostasis, it was an open-label study and therefore could not establish formal superiority over alternative strategies in terms of clinical efficacy or survival outcomes [8,19]. Previous phase 1 and phase 2 studies included randomized, placebo-controlled designs; however, these were conducted in healthy volunteers and were primarily intended to assess pharmacodynamic endpoints rather than clinical outcomes such as survival, bleeding control, or transfusion requirements [15,19]. Future research should prioritize adequately powered RCTs and pragmatic trial designs in real-world perioperative settings, including emergency surgery and major bleeding cohorts, to better reflect clinical practice. Another unresolved issue is the long-term safety profile and thromboembolic risk following ticagrelor reversal. Bentracimab has generally been well tolerated, with few reported infusion-related adverse effects [16-18]. However, follow-up durations in existing studies are relatively short, and thromboembolic events have been assessed in largely non-comparative analyses. Prospective studies with longer follow-up and standardized adjudication of thrombotic outcomes are needed to better define long-term safety. There is also a potential risk of ischemic events following reversal of antiplatelet therapy, particularly in patients with recent acute coronary syndromes [21]. In addition, future studies should systematically evaluate key clinical endpoints, including mortality, transfusion requirements, rebleeding rates, and thromboembolic complications.

Furthermore, there is a lack of comparative effectiveness data. Currently, management of ticagrelor-associated bleeding often relies on nonspecific approaches such as platelet transfusion, which have limited efficacy due to the reversible and circulating nature of ticagrelor [17]. Comparative effectiveness data for bentracimab, particularly against standard care strategies, remain limited. Well-designed randomized or pragmatic trials are needed to evaluate its performance in comparison with current standard-of-care approaches, including in clinically relevant subgroups such as patients with renal impairment or those receiving concomitant anticoagulant therapy.

Finally, real-world data from perioperative and bleeding registries represent an important area for future research. Trial populations do not fully capture the heterogeneity of clinical practice, including variation in patient characteristics, procedural urgency, timing of last ticagrelor dose, and long-term outcomes. Real-world evidence would complement randomized controlled trial findings and help inform practical perioperative protocols for ticagrelor reversal [11,21]. Such registries should incorporate standardized outcome measures, including clinical effectiveness, safety, healthcare utilization, and cost-effectiveness endpoints.

## Conclusions

Bentracimab is a promising targeted reversal agent for ticagrelor-related hemorrhage, providing rapid restoration of platelet function in critical clinical situations. Preliminary evidence, largely derived from non-comparative and short-term studies, supports its role in facilitating hemostasis and assisting emergency surgical procedures, with a favorable short-term safety profile.

However, important uncertainties remain, particularly regarding long-term safety, thrombotic risk, and comparative effectiveness, given the current reliance on non-comparative and short-term data. Further randomized trials and real-world evidence are required to better define its role in clinical practice.
